# On the Processability and Antibacterial Activity of Silver Nitrate Nanocomposites Manufactured by Stereolithography

**DOI:** 10.1021/acsomega.5c00030

**Published:** 2025-05-19

**Authors:** Ayberk Baykal, Onur Alp Aksan, Ahmet Yavuz Oral, Kaan Bilge, Nuray Kizildag

**Affiliations:** † Institute of Nanotechnology, 52962 Gebze Technical University, Kocaeli 41400, Turkey; ‡ Department of Materials Science and Engineering, Faculty of Engineering, Gebze Technical University, Kocaeli 41400, Turkey; § Faculty of Engineering and Natural Sciences, Materials Science and Nano Engineering, 52991 Sabanci University, Istanbul 34956, Turkey

## Abstract

This study investigates the level of multifunctionality that might be achieved with the implementation of silver nitrate (AgNO_3_) to UV-curable resins during stereolithography. To achieve that it sets improved mechanical response and antibacterial activity of the additively manufactured nanocomposites as two objectives to be achieved without disturbing the printing quality. The resins containing 0.1, 0.3, 0.5, 1, 3, and 5 wt % AgNO_3_ were mixed via high shear mixing under 6000 rpm. A desktop-scale SLA machine and a postcure UV device were employed for the manufacturing of nanocomposite specimens. The level of printing quality was evaluated by SEM analysis focusing on the layer-by-layer printing marks that were highly disturbed after 1 wt % particle loading. Potential chemical effects causing this disappearance were investigated with UV–visible spectroscopy and FTIR analysis. Results suggested that some of the provided UV energy was used for the conversion of silver cations of silver nitrate to AgNPs during the polymerization reaction. Such a reduction reaction was found to decrease the degree of monomer conversion with increasing particle amount, which was confirmed by the FTIR analyses. Results of mechanical tests assisted by a detailed fractographic analysis also confirmed that 1 wt % was a particle agglomeration threshold above which the mechanical response was highly deteriorated. The maximum mechanical performance with a 20% improvement in elastic modulus and yield strength values was noted for 0.5 wt % case. The antibacterial activity tests were then performed on 0.3, 0.5, and 1 wt % samples according to ISO 22196. The results suggested that antibacterial activity was maximum for 1 wt % particle containing nanocomposites. Hence, the aim of a stronger, antibacterial yet adaptable nanocomposite material design was achieved at 0.5 wt % silver nitrate loading.

## Graphical abstract

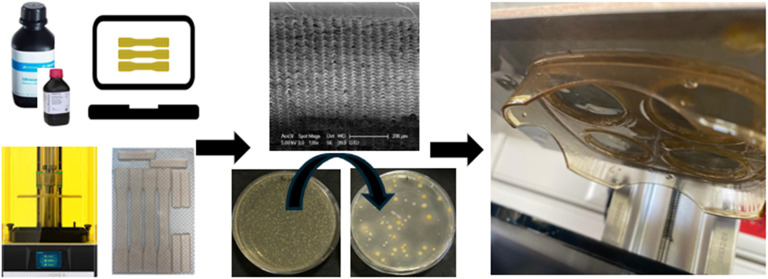

## Introduction

At the forefront of modern manufacturing technologies, additive manufacturing (AM) is a revolutionary technology that precisely constructs parts from 3D model data by joining materials layer upon layer, contrary to subtractive manufacturing. It provides a broader range of flexibilities in terms of material design, fast manufacturing, multimaterial options, waste reduction, high precision, and quality at low costs for several industrial sectors such as aerospace, electronics, telecommunication, biomedical, construction, mechanical, and defense parts.^1–6^ The key benefit of this innovative production method lies in its ability to create highly complex geometries with precision at a low cost. In certain situations, AM is the only feasible way to produce intricate components, while in others, it serves as a useful tool to minimize the number of separate parts that would otherwise need to be assembled. It also provides significant advantages during the prototyping stage, dramatically lowering both the time and expense required to develop and test multiple prototype versions for functionality, appearance, and performance. Additionally, AM can reduce the cost of producing customized items compared to traditional manufacturing techniques.^7^

The process begins with the creation of a 3D model of the object, which is then processed and divided into thin layers by using specialized software. The AM system constructs the object by printing each 2D layer sequentially, stacking them one on top of the other. This layer-by-layer approach results in the final 3D object produced by the printer.^8^ Various technologies, including selective laser sintering (SLS), fused deposition modeling (FDM), digital light processing (DLP), and stereolithography (SLA), have reached a well-established level of technological development. FDM uses a thermoplastic filament, while SLS relies on thermoplastic powder. On the other hand, DLP and SLA—classified as vat photopolymerization methods—operate by selectively solidifying a liquid resin through rapid photopolymerization. The final characteristics of the printed object can be easily adjusted by modifying the reactive resin formulations.^2,9–11^

Stereolithography, as a pioneering process within AM techniques, keeps pushing the boundaries of innovation in a number of sectors, from consumer products and healthcare to the automotive and aerospace industry, by making it possible to quickly prototype and produce detailed designs with unmatched precision and efficiency. Therefore, it is essential to comprehend the basic principles and applications of SLA in order to fully realize its value in manufacturing.^10–13^ Wider use of stereolithography is limited by the restricted availability of commercial resins with desired properties.^9^ The properties of the available resins can be modified by the addition of micro- and nanoparticles into the resins.^14,15^

Thanks to their versatile properties and wide range of applications, silver nanoparticles (AgNPs) embedded in polymer matrices have garnered growing interest. AgNPs are classified as nanomaterials with dimensions between 1 and 100 nm.^16^ At this scale, silver exhibits distinctive electrical, optical, and catalytic behaviors, which have prompted research into its use in targeted drug delivery,^17,18^ diagnosis,^19^ detection,^20^ and imaging.^21^ However, it is their remarkable antibacterial effectiveness that has drawn significant attention from both researchers and industry. AgNPs have demonstrated antimicrobial effects against numerous infectious and drug-resistant pathogens.^22^ The enhanced antibacterial performance of nanoscale silver^23^ has proven especially valuable in healthcare and medical fields, where it has been explored for integration into a wide range of products, such as surgical instruments, dental materials, catheters, wound dressings, as well as items used in food handling, apparel, and cosmetics.^24^

The incorporation of silver nanoparticles (AgNPs) into polymers is a specific topic of interest. AgNPs have attracted the interest of researchers due to their unique optical, electrical, and antibacterial capabilities, which make them promising candidates as additives in the preparation of polymer nanocomposites with functional properties. AgNPs’ antibacterial properties make them effective against bacteria, viruses, and other microbes. Silver nanoparticles are also nontoxic, nonallergenic, and highly stable. Their small shape and high surface area-to-volume ratio also help to explain their increased reactivity and functionality.^22,25,26^ In addition, the use of silver nanoparticles enhances the mechanical strength of polymer nanocomposites, making them suitable for various applications that demand durable and resilient materials, such as structural and automotive parts.^27,28^ Furthermore, silver nanoparticles have high electrical conductivity, which makes them useful for conductive coatings and electronics. In UV-curable polymers, where resistance to UV-radiation-induced deterioration is crucial, their UV-blocking properties also make them important. This property increases the longevity of materials and guarantees continued functionality in adhesives, paints, and optical components.^22,25,29^ UV-curable polymers, which are extensively used in additive manufacturing, can be modified by adding AgNPs so that the final printed items display antibacterial qualities.^30,31^ This is especially useful in healthcare applications, where bacterial development needs to be avoided.^29^ SLA technique enables the in situ synthesis of AgNPs within UV-curable resins without the need for additional processes, which are performed for the reduction of silver precursors into AgNPs in the polymer structure. The increase in the viscosity of resin after the nanoparticle addition and sedimentation of the nanoparticles in the resin limits the widespread use of the technique and calls for careful selection of the resin, nanoparticle concentration, and process conditions.^30^ On the other hand, the incorporation of silver nanoparticles in a polymer matrix can significantly affect the properties of the matrix.^32^ The advantageous chemical and physical characteristics of polymer nanocomposites containing AgNPs have already found widespread application in various fields. Stereolithography can further enhance this trend by offering design flexibility in the creation of 3D engineered structures with tailored shapes.^7^

Singh and Khanna were pioneers in synthesizing silver nanoparticles (AgNPs) within poly­(methyl methacrylate) (PMMA) using a one-step in situ approach. In their method, the polymer was first prepared, followed by the addition of a silver nitrate (AgNO_3_) solution in *N*,*N*′-dimethylformamide (DMF) to a PMMA solution also in DMF to form the nanocomposite. The incorporation of up to 10 wt % of silver nitrate resulted in improved thermal stability.^33^ Singh et al. developed PMMA/AgNPs nanocomposites using DMF as the solvent for both dissolving the polymer and dispersing the silver nanoparticles, with the entire process monitored via FTIR spectroscopy. PMMA was first dissolved in DMF, after which stock solutions of AgNPs were incorporated into the polymer solution. The resulting nanocomposite was analyzed by using transmission electron microscopy (TEM) and Fourier transform infrared spectroscopy (FTIR). TEM analysis at a 10% silver loading showed spherical Ag/PMMA particles with an average size of 24 nm and a narrow size distribution. It was also observed that the average diameter of AgNPs increased with increasing temperatures at higher silver concentrations. Additionally, as the concentration of the PMMA/AgNPs solution increased, the stretching vibration of the ester carbonyl (C=O) group shifted to lower wavenumbers.^32^ Siddiqui et al. synthesized PMMA/AgNP nanocomposites through an in situ radical polymerization method, during which the reduction of silver ions (Ag^+^) occurred simultaneously with the polymerization reaction. They studied the influence of silver nanoparticles on reaction kinetics by tracking conversion over time and analyzing the molecular weight distribution of the resulting polymer. FTIR results indicated that the incorporation of AgNPs into the polymer matrix was physical in nature without chemical bonding. The silver nanoparticles formed had average sizes between 37 and 47 nm, with broader size distributions seen at higher silver concentrations. The in situ generation of AgNPs was attributed to a radical mechanism involving chain transfer reactions. The presence of AgNPs reduced the efficiency of the initiator, leading to a slower reaction rate and a slight increase in the number-average molecular weight. Both the polydispersity and glass transition temperature of the polymer decreased with increasing AgNP content. It was also confirmed that AgNPs promoted the formation of polymer chains with fewer structural defects.^34^ Taormina et al. demonstrated the in situ formation of silver nanoparticles (AgNPs) from a uniform acrylic resin mixture containing well-dispersed silver salts—specifically silver acetate, silver acrylate, and silver methacrylate. During the stereolithographic printing process, these salts were reduced to metallic silver. A commercial stereolithography printer equipped with laser radiation was employed to both cure the resin layer by layer and simultaneously reduce silver ions to AgNPs. The resulting nanocomposite materials exhibited enhanced physical and mechanical properties compared with the unmodified resin. Notably, even at low concentrations of AgNPs, the mechanical performance improved significantly. The use of silver salts with carbon–carbon double bonds, like silver acrylate and silver methacrylate, enabled the development of a nanocomposite structure with minimal byproduct formation, as all reactive components actively participated in the 3D printing process.^7^ Valencia et al. explored the in situ formation of silver nanoparticles (AgNPs) from different silver precursors, namely, silver nitrate (AgNO_3_) and silver perchlorate (AgClO_4_), within rigid acrylic resins using stereolithography (SLA). Transmission electron microscopy (TEM) confirmed the successful formation of AgNPs less than 5 nm in size across all nanocomposites, imparting optical activity to the materials. A high concentration of AgClO_4_ led to a dense and well-dispersed distribution of nanoparticles throughout the resin, while lower precursor amounts (0.1%) resulted in more isolated agglomerations. The addition of AgNPs significantly reduced the material’s electrical resistivity compared to the unfilled resin. However, the use of the photoinitiator in the AgNP formation process reduced the overall degree of polymerization, which in turn negatively impacted the mechanical properties of the final nanocomposite.^31^ Integrating nanoparticles into curing processes presents opportunities to enhance the performance, efficiency, and functionality across various applications. However, resins containing nanoparticles are often unsuitable for vat photopolymerization methods, as the inclusion of rigid particles significantly increases the viscosity of the reactive mixture, making the process more challenging.^7^ Careful consideration of nanoparticle properties and their interactions with the curing system is essential to harness their full potential and avoid any undesired effects.^35^

As the stereolithography technique is a relatively new technique, there is limited literature about the polymer nanocomposites prepared via this technique. Originating from the existing state-of-the-art, this work aims to exemplify the usage of stereolithography as a fast and reliable way to obtain multifunctional nanocomposites of AgNO_3_ with improved mechanical response and antibacterial activity. To achieve that it employed a high shear mixing strategy followed by printing in a desktop-scale SLA machine and assessed the effects of six particle loading ratios such as 0.1, 0.3, 0.5, 1, 3, and 5 wt %. The processability and printability of the resin mixtures in conventional SLA machines were assessed with morphological analysis focusing on the disruptions on the 3D architecture of the printed parts due to nanoparticle presence. It then revealed the in situ effects of AgNO_3_ nanoparticles via UV–Visible Spectrometry and FTIR analysis, where first focused on the AgNO_3_ reduction mechanism and latter focused on the degree of conversion. The mechanical properties of the nanocomposite candidates were measured by tensile testing. Their contributions to the mechanical response were revealed by a detailed fractographic analysis. Lastly, the achieved level of multifunctionality was assessed by the antibacterial response of the nanocomposite solutions. Going beyond the state of art, this study aims to provide a complete assessment of the processability of multifunctional nanocomposites by material characterization assisted with visual failure analysis targeting the prevention of 3D architecture with employed nanoparticles. Moreover, it employs fractographic analysis to underline the active failure mechanisms to pave the way to a deeper understanding for further studies aiming to achieve multifunctionality.

## Experimental Details

### Materials

Ultracur3D ST 80, which is a technical material based on (meth)­acrylate resin for suggested SLA systems, was used as the matrix resin. It is a colorless tough resin with working wavelengths of 355, 385, or 405 nm. Silver nitrate (AgNO_3_) (Alfa Aesar Premion, 10858) was used as the precursor salt for in situ synthesis of AgNPs in the tough photocurable resin. Ethanol was used in the washing process.

### Methods

#### Preparation of the Formulations

Varying amounts of silver nitrate (0.1, 0.3, 0.5, 1, 3, and 5 wt % based on resin weight) were mixed into the resin by using a high-speed mixer at 6000 rpm for 20 min. Following the mixing process, the resulting nanocomposite resin formulations were allowed to stand for 10 min to facilitate the removal of air bubbles generated during mixing.

#### Preparation of the Polymer Nanocomposite Specimens

The models for the specimens were designed by using Freecad CAD software and then converted to a g-code file to be processed by the SLA printer. For composite specimen production, an Anycubic Photon Mono X 4K SLA machine was used. The neat resin and composite resin mixtures were poured into the pool of the SLA printer. The wavelength of the UV light was set as 405 nm, and the curing time for a single layer was set to 4 s. Each specimen consisted of 60 layers with a layer height of 50 μm. Neat and nanocomposite specimens were printed onto the SLA printer platform with a stacking direction along the specimen thickness, which underwent subsequent structural and functional characterizations. The printed specimens were detached from the printer platform and placed in the Anycubic wash and cure machine for washing and postcuring processes. A 10 min washing with ethanol was applied to remove the unreacted resin. After the washing, postcure was applied for 20 min under 405 nm UV radiation to complete the photopolymerization process, partially inhibited by the addition of the silver salt.^26^ Four dog-bone-shaped specimens and six rectangular specimens were prepared with each composition for characterization. A schematic showing the printing steps via SLA technique is presented in Figure 1.

**Figure 1:**
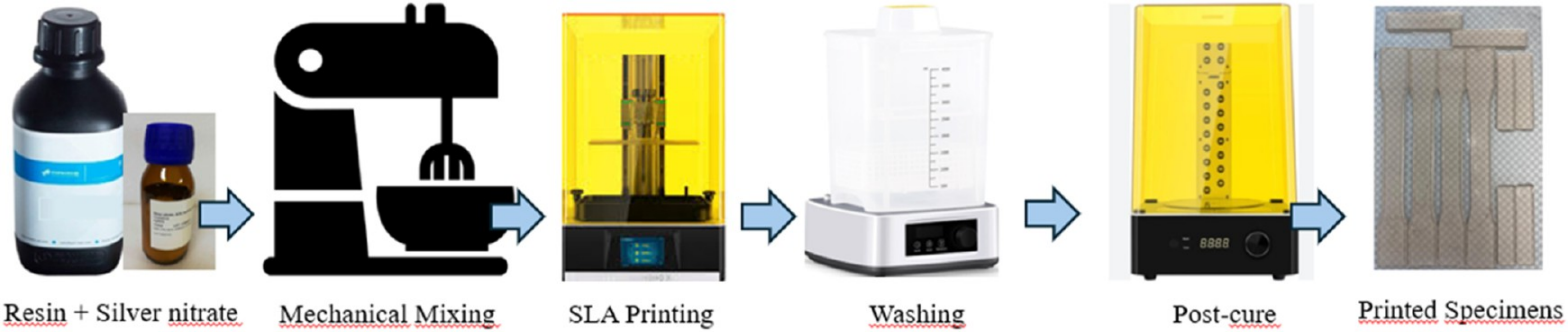
Schematic showing the steps of the SLA process.

#### Characterization

Silver nitrate was added into an acrylic-based photopolymer resin in varying mass ratios, ranging from 0.1 to 5%, and the effects of the silver nitrate addition on the printing process, curing mechanism of the resin, and properties of the composite specimens were investigated. The processability of the composite resins was assessed along with the effect of silver nitrate addition on the periodicity of the printed specimens. The nanocomposite samples were characterized, and their properties were compared to those of the neat specimens obtained in the same way without adding any silver nitrate to the resin formulation. Scanning electron microscopy (SEM), energy-dispersive X-ray spectroscopy (EDX), Fourier transform infrared spectroscopy (FTIR), and UV–visible spectroscopy (UV–vis) were used for characterization. Tensile tests were conducted to determine the mechanical properties. Additionally, composite specimens containing silver nanoparticles were tested for their antibacterial activity. Resin mixtures were denominated with R and silver nitrate contents (R-0.1, *R*-3̅, and so), while the printed specimens were denominated with PC and silver nitrate contents (PC-0.1, PC-3, and such).

#### Scanning Electron Microscopy (SEM)

The surface morphology and fractured surfaces of the specimens containing different amounts of silver nitrate were analyzed via SEM. A Philips XL30 SFEG scanning electron microscope was used to take SEM images. The fractured surfaces of the specimens were compared to each other in order to understand the fracture mechanism.^36^

#### Energy-Dispersive X-ray (EDX) Analysis

Energy-dispersive X-ray spectroscopy (EDX) was used to identify and measure the elemental composition of a selected specimen. A Philips XL30 SFEG scanning electron microscope was used to take EDX images.^36^

#### Fourier Transform Infrared Spectrometer (FTIR)

FTIR was utilized to investigate the curing of the resin and resin mixtures. A PerkinElmer Spectrum 100 FTIR Spectrometer was used to record the infrared absorption spectra of neat and nanocomposite resins and specimens in the range from 4000 to 750 cm^–1^ with a resolution of 4 cm^–1^. Sixteen scans were taken for each specimen and averaged to produce FTIR spectra.

The degree of conversion (DC) was determined by measuring the decrease in the intensity of the methacrylate (C=C) stretch absorption band at 1636 cm^–1^. The baseline of the FTIR spectra was corrected and then normalized to the carbonyl peak at 1720 cm^–1^, which remains unaltered during the curing process in order not to account for the sample thickness.^37^ The peak heights were measured in relation to the baseline, which was traced by connecting the troughs of each peak in the wavenumber range from 1580 to 1800 cm^–1^.^38^ DC is calculated based on the following formula:

$$\mathrm{degree}\;\mathrm{of}\;\mathrm{conversion}\;\left(\mathrm{DC}\right)\;\left(\%\right)=100-\frac{\mathrm{Abs}\;\left(1636\right)\;\mathrm{of}\;\mathrm{the}\;\mathrm{polymer}}{\mathrm{Abs}\;\left(1636\right)\;\mathrm{of}\;\mathrm{the}\;\mathrm{resin}}\times 100$$

where Abs are the height of the absorption band. DC is determined by subtracting the residual percentage of aliphatic C=C from 100%.

#### UV–Visible Spectroscopy (UV–vis)

UV–vis was employed to investigate the curing of the resin and resin mixtures and the formation of silver nanoparticles. A Shimadzu 3600i Plus spectrophotometer was used to obtain UV–visible spectra of neat resin and printed specimens. A scan rate of 10 nm/s was used to monitor the absorbance intensity within the wavelength range between 350 and 700 nm.^36^

#### Mechanical Tests

Tensile mechanical tests were performed using an Instron 5569 testing machine with a load capacity of 15 kN. The samples for tensile tests were prepared and tested according to the ASTM D638 Type-I standard.^39^ The sizes of all specimens were 165 mm × 13 mm × 3 mm (length × width × thickness). Speed was set to 5 mm/min. Three specimens were tested for each composition to determine their ultimate tensile strength.

#### Antibacterial Activity

Antibacterial activity of the printed specimens containing 0.3, 0.5, and 1 wt % silver nitrate against S. aureus was determined according to ISO 22196 standard and reported in comparison to the control sample.^40^ The antibacterial tests were repeated thrice.

## Results and Discussion

### Processability of the Composite Resins

The incorporation of nanomaterials into printable resins has been shown to influence both the printability and processability of the resin while adding functional properties to the final printed object. Literature indicates that stereolithography can be significantly impacted by the presence of nanomaterials, as these particles may interact with the light source through absorption or scattering or interfere with components of the resin formulation. The extent to which nanoparticles affect the printing process is closely linked to their intrinsic properties and the extent to which they are dispersed within the resin. For example, Jonušauskas et al. demonstrated this phenomenon through plasmon-assisted 3D microstructuring of gold-doped polymers. They observed that the printed line width was highly sensitive to the concentration of gold nanoparticles (AuNPs). As the AuNP concentration increased, the size of the printed features also grew, while lower concentrations resulted in narrower line widths. These findings suggested that AuNPs could partially substitute for the photoinitiator. The underlying mechanism was attributed to the plasmonic effect of the gold nanoparticles.^6,8,41^ Peng et al. reported that incorporating CdSe quantum dots into a photocurable resin led to enhanced printing resolution, while also causing a decrease in monomer conversion during the process.^42^ Liu et al. developed a technique to produce 3D printed structures containing silver nanowires to enable electrical conductivity. A decrease in line width was noted, but it was not further investigated.^43^ Nanoparticles can interact with light excitation through either single-photon absorption (1PA) or two-photon absorption (2PA), which can disrupt the effective formation of photogenerated radicals.^42,44^ Additionally, nanoparticles (NPs) can directly hinder polymerization when radical species come into contact with their surfaces.^45^ This issue becomes more complex when NPs agglomerate within the resin. Plasmonic NPs, in particular, exhibit altered optical properties, depending on their colloidal state. Agglomeration-induced plasmonic coupling can significantly shift the plasmon resonance. Achieving uniform dispersion of NPs in the resin at high concentrations without agglomeration is a challenging task due to the attractive forces between NPs that promote agglomeration. Notably, agglomeration can occur even before the printing process begins, such as when NPs are added to the uncured resin.^46,47^ Since agglomeration can affect the properties of the NPs,^46^ it can impact not only the final properties of the printed structures but also interfere with the printing process itself. Specifically, scattering or absorption of the curing laser light by nanoparticle agglomerates must be carefully considered. BASF ST45 and ST80 resins were selected for this study in order to compare the effects of the addition of silver nitrate to two different resins with different characteristics. It was not possible to print specimens from composite resins prepared by using BASF ST45 resin. Due to the higher viscosity of the resin, it was difficult to disperse the silver nitrate in the resin, and intense bubble formation was the case during mechanical mixing. In addition to the negative effects of the agglomeration explained above, the bubbles in the resin can also impact the quality of the printed parts by affecting the UV light penetration into the resin in different ways. Bubbles dispersed throughout the resin, if not removed before printing, can interfere with the UV light and cause uneven curing or incomplete polymerization in regions where the UV light intensity is reduced, as a result of which the printed part may exhibit inconsistencies in surface finish, mechanical properties, or dimensional accuracy. Bubbles can absorb UV light, reducing the amount of energy available for curing the surrounding resin. This absorption can lead to undercuring or inadequate cross-linking of the polymer, resulting in weak or brittle regions within the printed part. Bubbles may refract UV light as it passes through the resin, altering its direction and intensity. This refraction can cause distortion or misalignment of the cured layers, affecting the overall dimensional accuracy and geometric fidelity of the printed object. In regions where bubbles are densely clustered or located close to the building platform, they can create shadowing effects that block UV light from reaching the underlying layers of resin. This can result in incomplete curing or adhesion between layers, leading to delamination or structural defects in the printed part.^48,49^

The photographs of the composite resins prepared using BASF ST 80 resin and the printed specimens are presented in Figure 2. Silver nitrate is white in color, and it does not affect the color of the resin when added into it. But as the silver nitrate is exposed to heat, and/or UV light, reduction of silver ions in the silver nitrate takes place, as a result of which silver nanoparticles form. With the progress of the reduction reaction, color changes from white to yellow and then to brown occur, which show the formation of silver nanoparticles.^36,50^ While the color change is taken as a sign of successful formation of silver nanoparticles in the literature,^51–54^ the resin mixtures need to be protected from heat and sunlight very well before the SLA printing process as the color change may negatively affect the printing process. It could be possible to print the composite formulations with silver nitrate content up to 5 wt % despite the dramatic color change, as most of the color change occurred during or after the printing process. The residual composite resins were not possible to reuse after the printing was complete, as intense reduction took place during the process.

**Figure 2:**
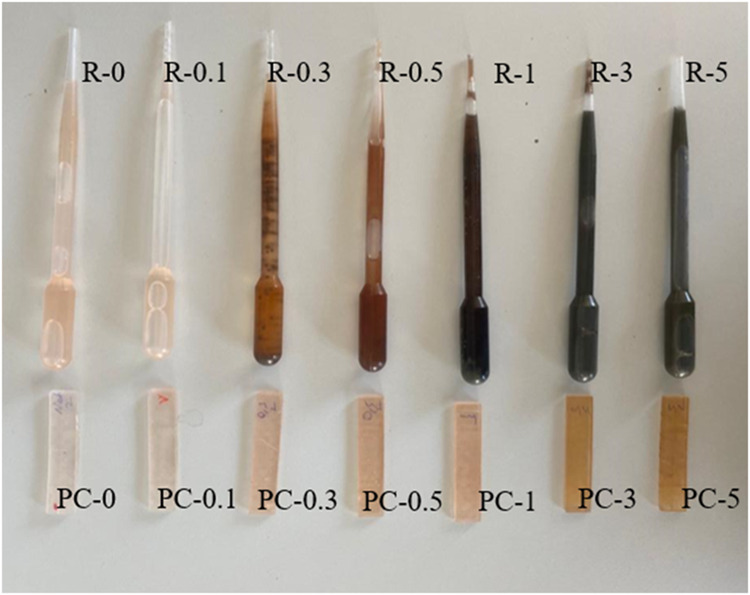
Photographs showing the neat and composite resins and printed specimens.

The frequency at which each layer of the 3D object is printed is referred to as the printing periodicity in SLA printing. Although the layer height can vary depending on the SLA printer and process parameters, the common values fall between 25 and 100 μm.^55^ The printing speed, resin viscosity, and other variables can also have an impact on printing periodicity. While a quicker printing speed could cut down printing time, it might also have an impact on print quality. Comparably, a resin with a lower viscosity may speed up printing but provide less accurate details.^56^ Such an effect was clearly visible on the cross-sectional views of printed samples, where a repetitive printing pattern was visible (Figure 3).

**Figure 3:**
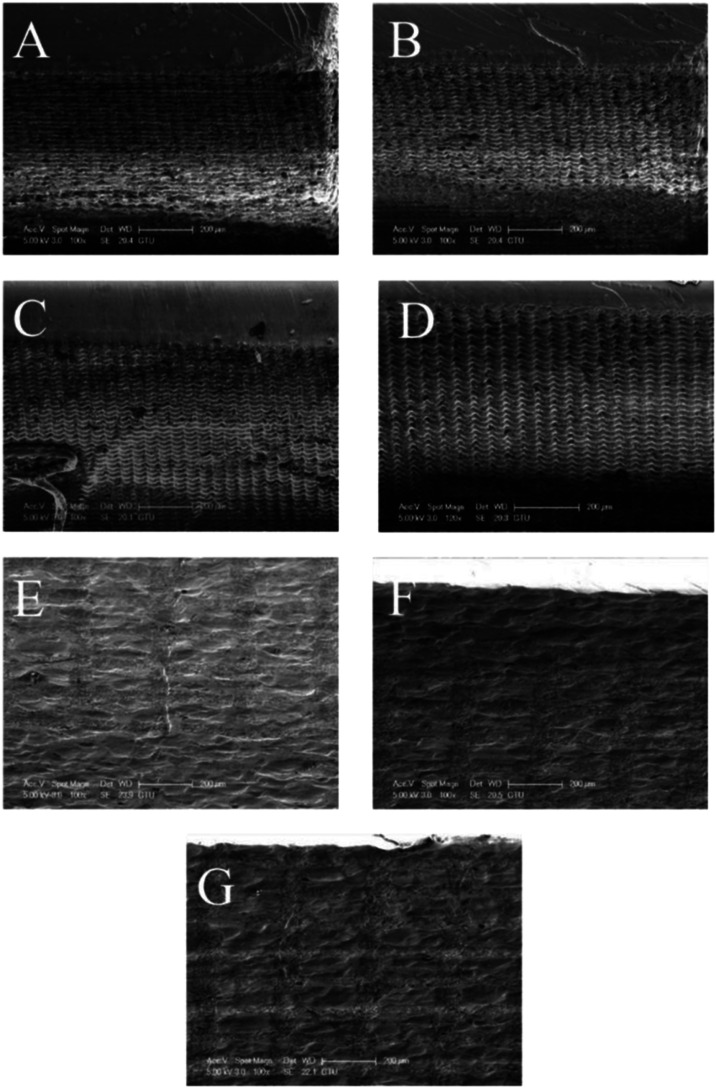
SEM images of (A) neat and composite specimens with (B) 0.1, (C) 0.3, (D) 0.5, (E) 1, (F) 3, and (G) 5 wt % AgNO_3_.

For samples containing 0.1 wt % (Figure 3B), 0.3 wt % (Figure 3C), and 0.5 wt % (Figure 3D) AgNO_3_, the nature of the printed layers was similar to neat samples (Figure 3A). This suggested that up to 0.5 wt % particle presence, the color of the resin solution enabled effective printing. However, above this ratio, the architectural pattern on the samples was almost completely lost (Figure 3E–G). It can therefore be suggested that preset manufacturing settings associated with optimum UV-curing and maximum detail efficiency were disturbed with the addition of AgNO_3_ particles above 0.5 wt %.

### Energy Distributed X-ray (EDX) Analysis

The EDX spectrum, obtained using spot profile mode from highly concentrated areas of AgNPs on the SEM image, exhibited peaks corresponding to Ag atoms along with signals of (N) atoms, which offered insights into the distribution of particles in associated polymer matrix.^57,58^ Depicted region from the PC-1 sample (Figure 4A) suggested that a homogeneous particle distribution (Figure 4B) was obtained. The plot of only the Ag element (Figure 4C) was used to estimate the in situ particle size distribution (Figure 4D). Results suggested that at PC-1 the average particle size was 630 nm with a mode value around 400 nm. Homogenized distribution along with measured average size value suggested that nanoparticle agglomeration started at this particle loading but did not yet reach a percolation threshold above which larger particulates (several micrometers) would form.

**Figure 4:**
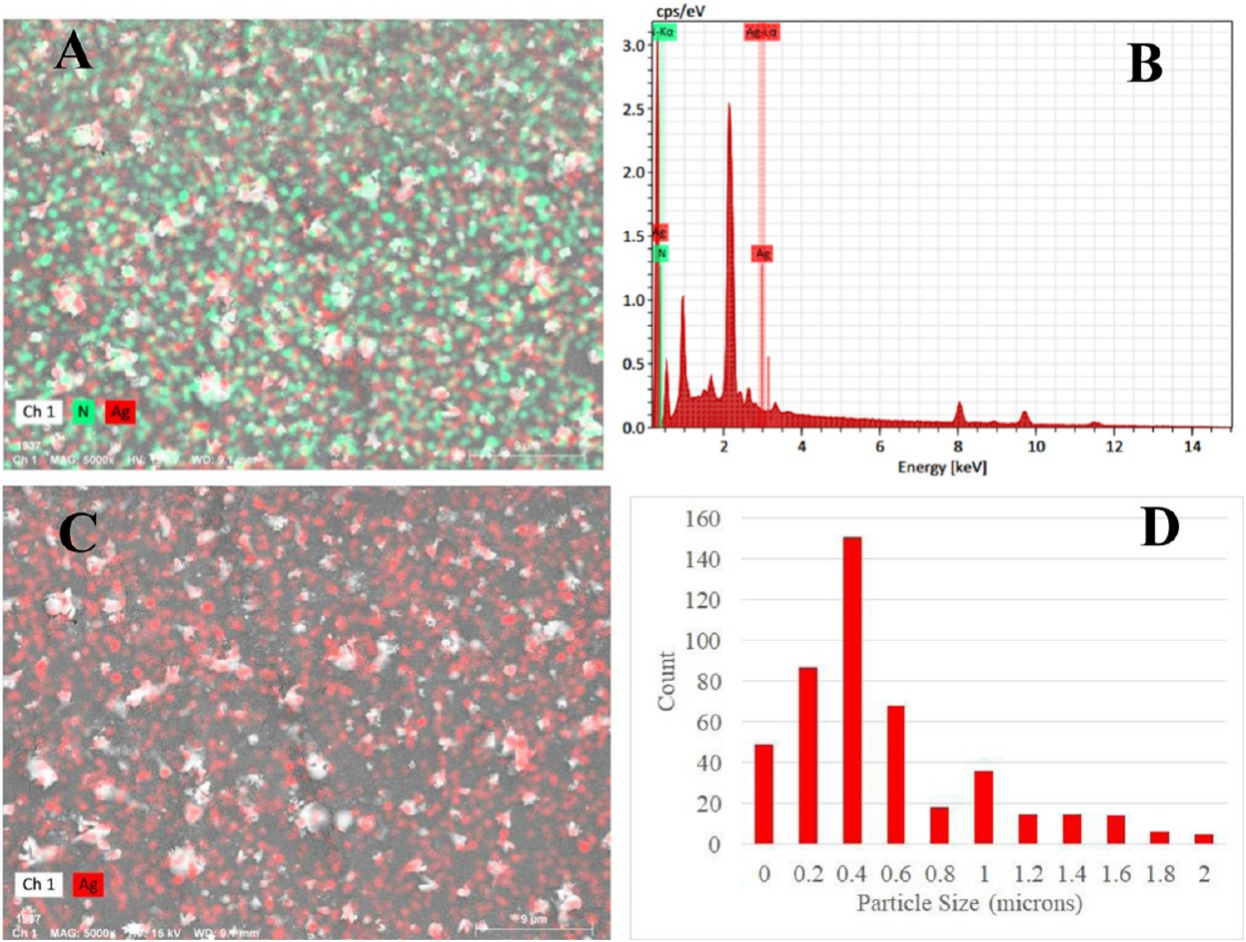
(A) EDX mapping, (B) EDX analysis of printed specimen containing 1 wt % AgNO_3_, (C) EDX mapping of Ag, and (D) particle size distribution of AgNPs.

### Fourier Transform Infrared Spectroscopy

FTIR is one of the most common methods that is used to determine the curing of photocurable resins. The stretching vibrations of carbon–carbon double bonds involved in polymerization are detected and compared to evaluate the curing.^59^ Figure 5A shows the FTIR spectra of the neat and composite resins, while Figure 5B shows the FTIR spectra of the neat and composite specimens after postcure treatment is applied.

**Figure 5:**
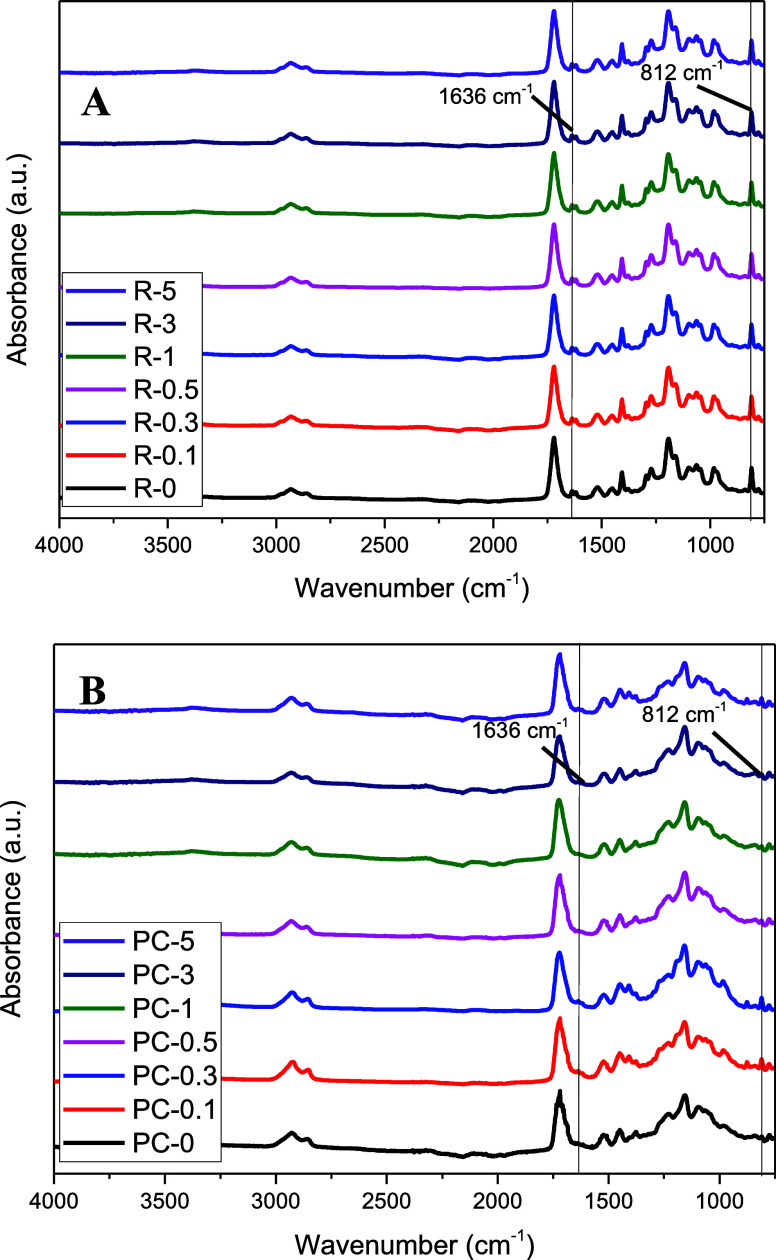
FTIR spectra of (A) neat and composite resins and (B) neat and composite specimens.

As no shifts were observed in the wavenumbers of the peaks, FTIR data showed that the incorporation of AgNPs in the polymer matrix was physical without the formation of any covalent bonds. In FTIR spectra, it is seen that the absorption bands of the acrylate group (C=C) at 1636 and 812 cm^–1^ significantly reduced after the curing treatment, as the resin monomers reacted with each other through the double bonds during photopolymerization (curing).^59,60^

To determine the degree of conversion (DC) in resins based on methacrylates, the mid-infrared spectral (MIR) region is frequently used. In the mid-infrared (MIR) region, the degree of conversion (DC) is determined by measuring the decrease in intensity (or area) of the methacrylate (C=C) stretch absorption band at 1636 cm^–1^ as the methacrylate monomer undergoes polymerization. The determination of DC is simplified when the material has a stable absorption band whose intensity remains unchanged during the polymerization. This stable band is used as an internal standard for normalization, eliminating the need to account for sample thickness.^37^ In this study, DC was calculated by comparing the absorbance peak corresponding to the aliphatic carbon–carbon double bond (1636 cm^–1^ peak height) to that of the carbonyl peak (1720 cm^–1^ peak height), following the methods outlined in a previous study. The peak heights were measured relative to the baseline, which was drawn by connecting the troughs of each peak within the wavenumber range of 1580 to 1800 cm^–1^.^37,38^ The FTIR spectra in the wavenumber range from 1580 to 1650 cm^–1^ after baseline correction and normalization are presented in Figure 6. DC values calculated are presented in Table 1.

**Figure 6:**
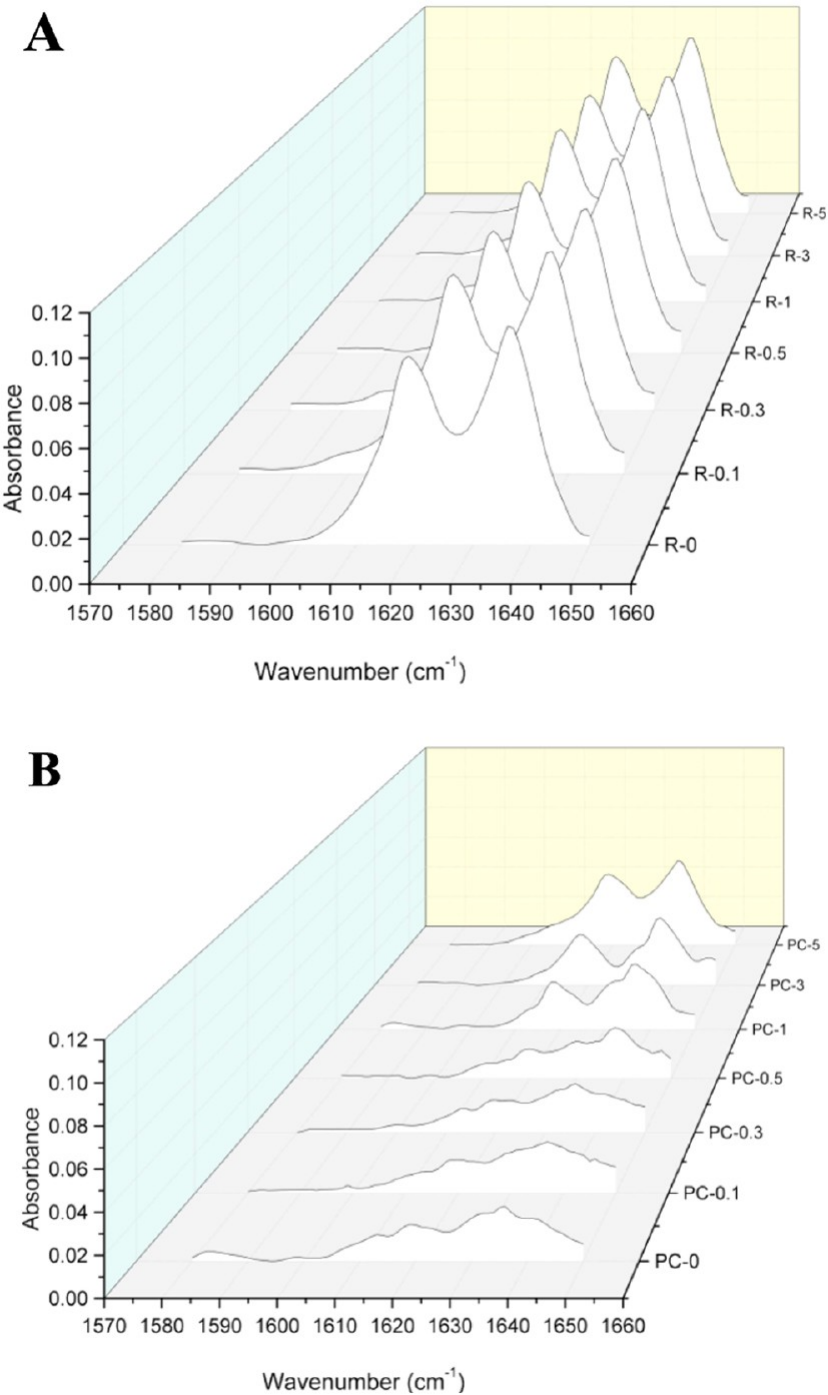
FTIR spectrum of (A) neat and composite resins and (B) neat and composite specimens showing the decrease in the intensity of aliphatic C=C bond with the photopolymerization..

**Table 1:** Intensity of Absorbance of the Aliphatic (C=C) Peak and the Degree of Conversion (DC) Percentages

	absorbance (at 1636 cm^–1^)		
silver nitrate content	R	PC	DC (%)
0	0.100	0.026	73.78
0.1	0.108	0.026	76.79
0.3	0.103	0.026	74.56
0.5	0.106	0.029	72.96
1	0.110	0.039	64.47
3	0.107	0.042	60.54
5	0.110	0.056	49.46

For UV-curable resins, the influence of certain additives on curing is reported in literature.^61^ Degree of conversion was affected by the presence of silver nitrate in the photocurable resin. While DC slightly increased with the addition of lower amounts of silver nitrate, a significant reduction in DC was the case with the increase in silver nitrate content likely due to the absorbance of some part of the UV light by the silver nitrate and consequent decrease in the number of monomers taking part in the photopolymerization.

### UV–Visible Spectroscopy (UV–vis)

UV–visible spectra of neat resin and composite resins with 0.1, 0.3, and 0.5 wt % silver nitrate are presented in Figure 7A, while the UV–vis spectra of the printed neat and composite specimens with different contents of silver nitrate are presented in Figure 7B. The resins containing 1 wt % and higher amounts of silver nitrate were very dark colored, so it was not possible to test these resins because of their very high absorbances.

**Figure 7:**
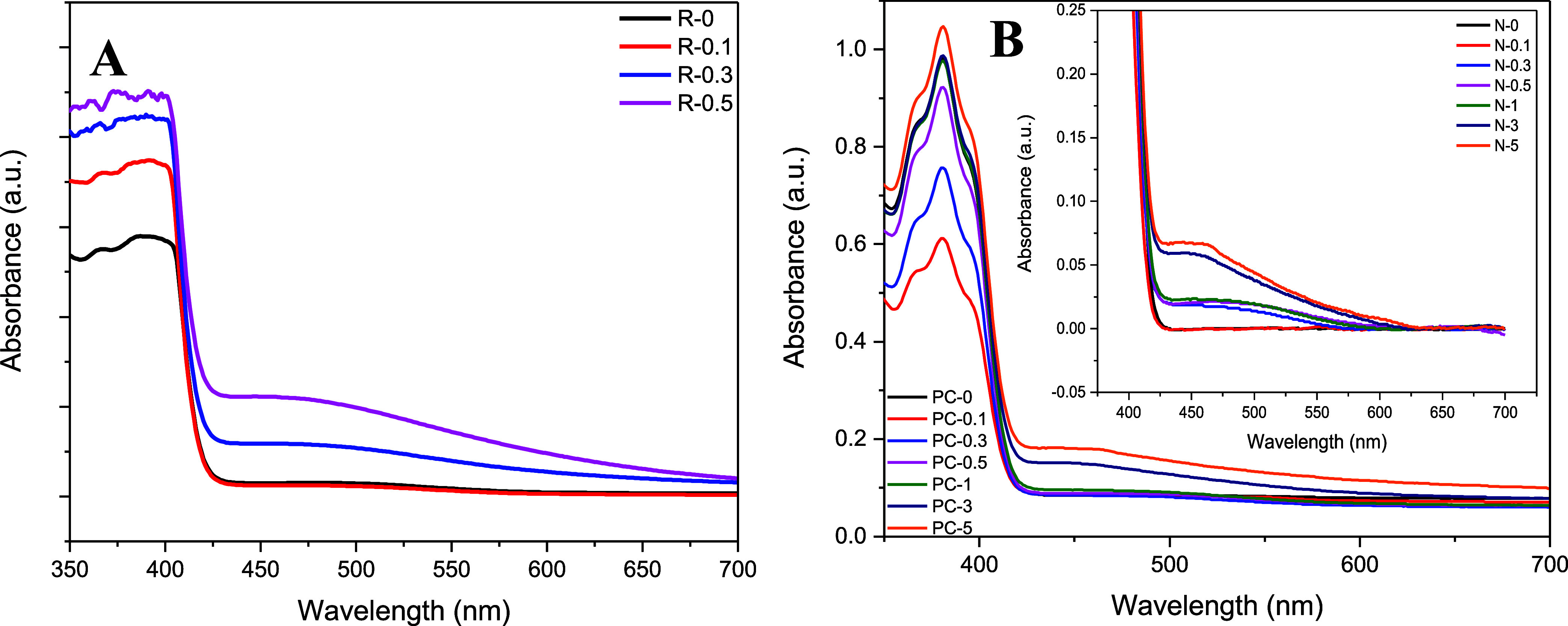
UV–visible spectra of (A) neat and composite resins and (B) printed neat and composite specimens.

The high absorbance of the resin between 350 and 430 nm increased with silver nitrate addition and reduction of silver nitrate, resulting in the color change and consequently changes in absorbance characteristics. The absorbance of the resin between 350 and 430 nm decreased after the specimen preparation due to photopolymerization taking place during the SLA and postcure treatment. Although not a very high correlation was observed between the absorbance values and silver nitrate content, a lower amount of silver nitrate addition was observed to increase the degree of conversion (lower absorbance values) in parallel with the FTIR results, while the effect on curing was not very prominent at higher loadings. Although some variations were observed with regard to curing with the silver nitrate content, the curing was not completed in any of the specimens in parallel with the results by Taormina et al.,^7^ who reported an incomplete polymerization reaction according to DSC analysis in the presence of silver salts as AgNP precursors. The appearance of the peaks between 425 and 600 nm with a maximum peak of around 450 nm for the silver nitrate containing samples showed the formation of individual silver nanoparticles in the size range of approximately 70 nm in the resin mixtures.^36,62^ The intensity of the peak generally increased with the increase in silver nitrate content.^36^ UV–visible spectroscopy confirmed the conversion of silver cations from silver nitrate to silver nanoparticles (AgNPs) as a result of the reduction process occurring concurrently with the polymerization reaction.^31,63,64^ The intensity of the UV radiation used in the SLA process was sufficient to trigger the chemical reduction, allowing for the in situ formation of AgNPs during the printing process, which differed from the behavior observed during printing with DLP.^7^

### Mechanical Properties

Results of the mechanical test performed for additive-manufactured composite samples are collated in Table 2. Initial attention was given to the relationship between Young’s modulus (E) and strain to failure (ε_max_) values measured from tensile tests (Figure 8A). Neat resin had an average E value of 804 MPa and an ε_max_ value of 10%; hence, it can be classified as a ductile resin with significant plastic deformation after polymer yielding. Implementation of AgNO_3_ particles seemed to increase the E value up to a threshold point captured in the PC-0.5 case. Parallel to that, the ε_max_ values remained rather constant between 7.2 and 7.5% up to 0.5 wt % silver nitrate addition. After this weight fraction, both E and ε_max_ began to diminish. This observation suggested that after 0.5 wt % silver nitrate addition, the AgNPs lost their ability to act as efficient stiffening agents, and agglomerations formed reduced the ε_max_ value and hence the ductility of the matrix. The reduction in E value can also be attributed to the loss of degree of conversion that was especially visible in the PC-5 case with even lower E values than the neat samples. Measured yield strength values (Figure 8B) suggested an increasing trend up to 0.3 wt % AgNO_3_ addition, after which it gradually decreased. It was previously discovered that the yielding event in ductile resin samples manufactured by SLA was related to the printed building blocks forming a single printed layer.^65,66^ Hence, the combination of elastic modulus and yield strength trends suggested that AgNO_3_ particles were effectively situated inside such building blocks up to 0.3 wt %. At around 0.5 and 1 wt % silver nitrate loadings, the particles began to get situated in-between such building blocks, and such effect became much clearer after 1 wt % silver nitrate loading due to large-size agglomerations.

**Figure 8:**
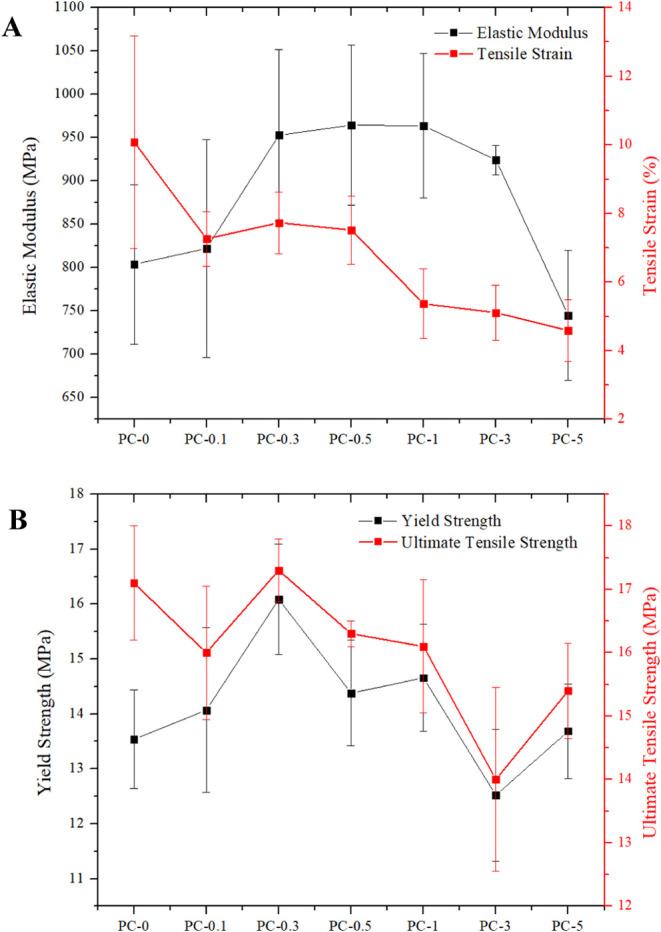
Mechanical properties of the printed composite specimens: (A) Elastic modulus and tensile strain variations. (B) Yield strength and ultimate tensile strength variations.

**Table 2:** Mechanical Properties of the Printed Neat Specimens and Composite Specimens

samples	elastic modulus (MPa)	yield strength (MPa)	ultimate tensile strength	strain at failure
PC-0	804 ± 92	13.5 ± 0.9	17.1 ± 0.9	10.1 ± 3.1
PC-0.1	822 ± 126	14.07 ± 1.5	16.0 ± 1.1	7.3 ± 0.8
PC-0.3	953 ± 99	16.1 ± 1.1	17.3 ± 0.5	7.7 ± 0.9
PC-0.5	964 ± 92	14.4 ± 0.8	16.3 ± 0.2	7.5 ± 1
PC-1	963 ± 83	14.7 ± 0.9	16.1 ± 1.0	5.4 ± 0.9
PC-3	924 ± 17	12.5 ± 1.2	14.0 ± 1.5	5.1 ± 0.8
PC-5	745 ± 75	13.7 ± 0.9	15.4 ± 0.8	4.6 ± 0.7

The traces of this effect were visible on the fracture surfaces of the failed tension samples (Figure 9). In the case of neat samples (Figure 9A), the fracture surface was rather smooth and contained river line patterns and ridges. The formation of river line patterns can be attributed to crack propagation from the boundaries of building blocks, whereas the ridges occur when multiple crack fronts coincide due to either collective failure of building blocks or their role as a crack generation locus.

**Figure 9:**
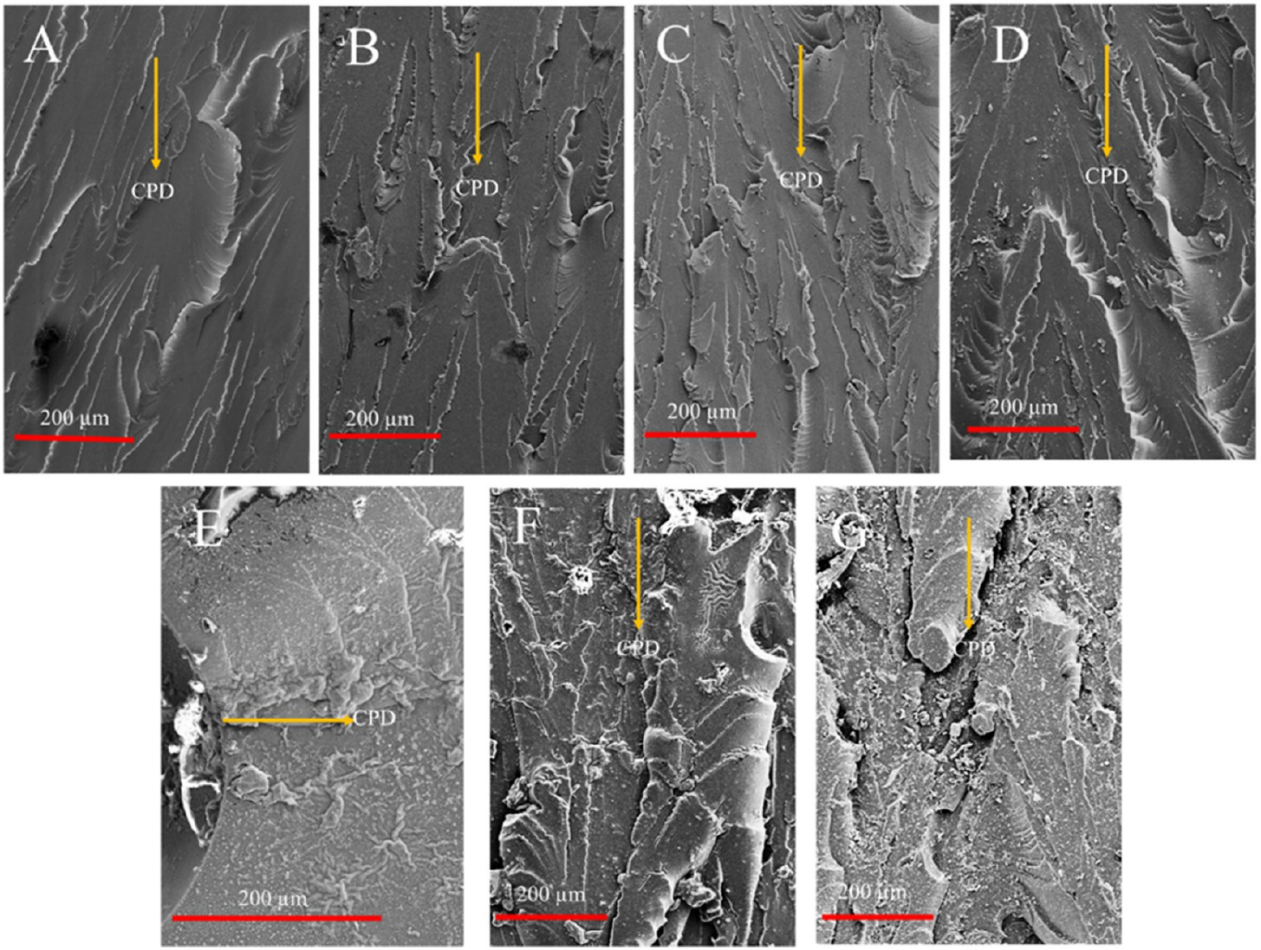
SEM images of the fractured surfaces of the printed (A) neat and composite specimen with (B) 0.1, (C) 0.3, (D) 0.5, (E) 1, (F) 3, and (G) 5 wt % AgNO_3_.

The addition of 0.1 wt % AgNO_3_ did not seem to cause drastic changes in fracture surface morphologies (Figure 9B). When the particle amount increased to 0.3 wt %, roughness changes on the fracture surfaces (Figure 9C) were visible. The size of the ridge was significantly increased, and ridges contained perpendicular crack propagation marks. This suggested that the presence of 0.3 wt % AgNO_3_ particles significantly strengthens individual building blocks by causing them to fail collectively. However, excess AgNO_3_ particles may cause additional cracking in the samples. In the case of 0.5 wt % loading, the overall appearance of the fracture surface was somewhat like PC-0.3 with only difference of visible AgNO_3_ agglomerations near the ridges (encircled region in Figure 9D). This suggests that local particle agglomerations began to occur after this particle loading level. The effect of such agglomerations became apparent on the fracture surfaces of PC-1 samples (Figure 9E). The failure in the investigated sample was due to a large and localized particle agglomeration around an air bubble, which majorly altered the failure around it. Both the river line and ridge formations were avoided, and the crack propagation direction was shifted. The diminished roughness of the fracture surface along with the perpendicular shift suggested a faster crack propagation. Although such major effect of agglomerations was not present in any other samples, such discussion is of importance to underline that AgNO_3_ reduction may have caused localized porosities. The fracture surfaces of PC-3 (Figure 9F) and PC-5 samples (Figure 9G) were again similar to PC-0.5 with larger-particle agglomerates being more visible, which were the causes of strength reduction.

Overall, the mechanical property measurements suggested that strength and modulus improvements achieved with AgNO_3_ are minor with respect to other types of similarly multifunctional nanoparticles such as TiO_2_ ^67^ or particles like SiO_2_ ^68^ whose main function is mechanical. However, when compared with similar works^7,31^ using AgNO_3_ as the nanoparticle type but applying different dispersion approach achieved improvements with a relatively high ratio of particles is superior. This makes applied simple high shear mixing advantageous. From the perspective of this study, it can be concluded that up to 1 wt % particle loading ratio, their presence did not negatively affect the mechanical response while being efficiently processable and having multifunctional characteristics. Failure analysis also suggested that with the adaptation of more sophisticated mixing methods along with high shear mixing, the particle amount can be increased, potentially increasing the mechanical performance.

### Antibacterial Properties

Nanoparticles offer opportunities for functionalization and surface modification, allowing for the incorporation of specific functionalities or properties into UV-curable polymers. Nanoparticles can introduce functionalities such as antimicrobial activity, flame retardancy, and self-cleaning properties, expanding the range of applications for UV-curable materials. AgNPs have demonstrated antibacterial activity against a range of both Gram-positive and Gram-negative bacteria. While the precise mechanism behind their bactericidal effect is not yet fully understood, current experimental studies suggest that the physicochemical properties of AgNPs, such as their size and surface characteristics, enable them to interact with or even penetrate cell walls or membranes, thereby directly affecting intracellular components.^22^ AgNPs serve as a reservoir and provide Ag^+^ continuously, which imparts the antibacterial property to polymers.^69–73^

The antibacterial efficiency of stereolithographically printed composite specimens containing silver nanoparticles was tested against S. aureus. Pathogenic bacterial strains of Gram-positive S. aureus were used to evaluate the antibacterial activity by calculating colony-forming units (CFUs). Figure 10 shows the pictures of agar plates with control samples and composite specimens containing 0.3, 0.5, and 1 wt % silver nitrate after 24-h incubation and depicts the viability loss of bacteria. Quantitative analysis based on CFUs (Figure 11) indicated that significant bacterial activity was obtained with the introduction of silver nitrate into the UV-curable resins. While negligible antibacterial activity was observed for the control sample, the CFUs for the composite specimens containing 0.3, 0.5, and 1 wt % silver nitrate were calculated as 3.08, 2.72, and 2.11 log, respectively, all of which corresponded to over 99.5% antibacterial efficiency in parallel with the study by Cui et al.^73^ Compared to Billings et al., who observed an antibacterial activity for the composite specimens containing 5 wt % ZnO,^74,75^ antibacterial activity was observed at much less additive loading.

**Figure 10:**
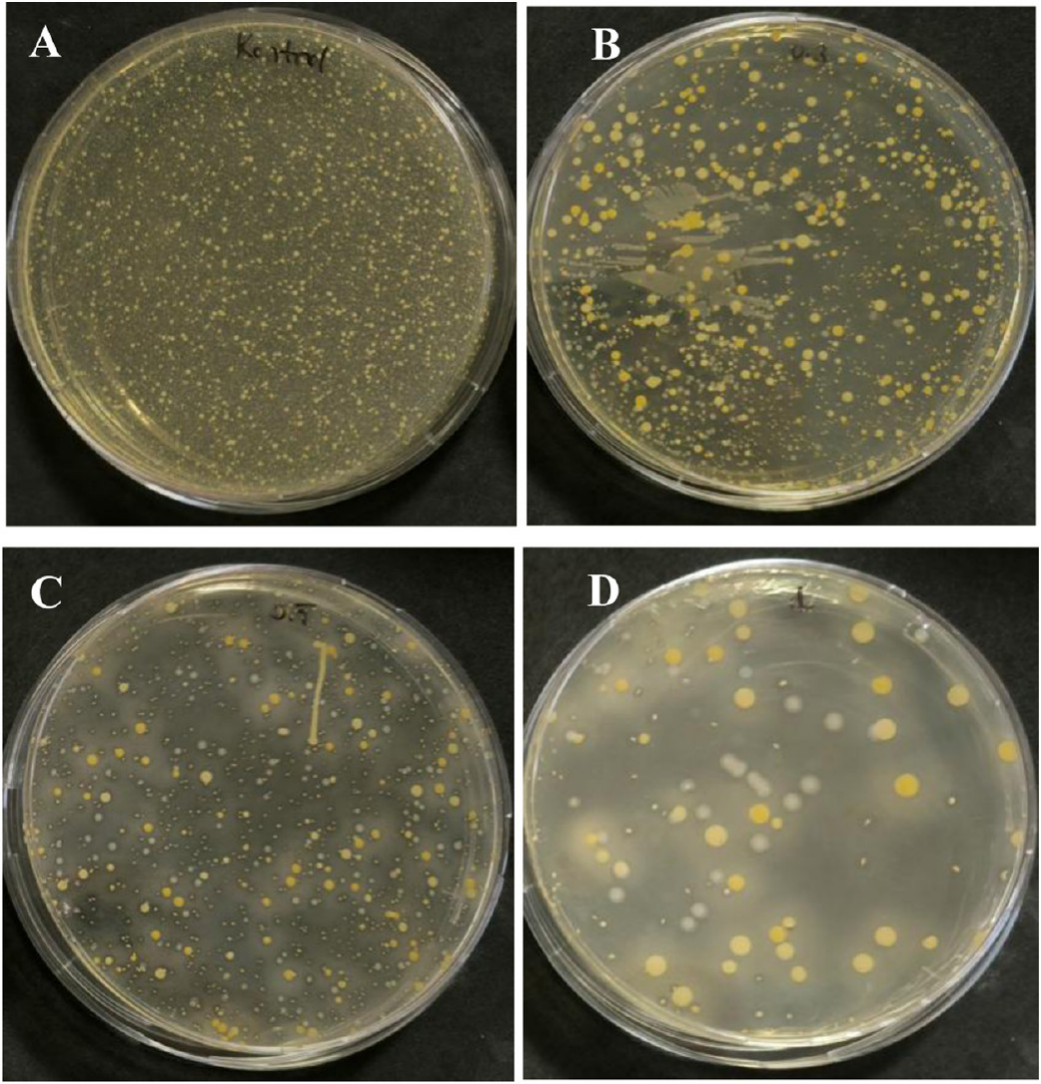
Pictures of agar plates with (A) control sample, and composite specimens containing (B) 0.3 wt %, (C) 0.5 wt %, and (D) 1 wt % silver nitrate, after 24 h incubation.

**Figure 11:**
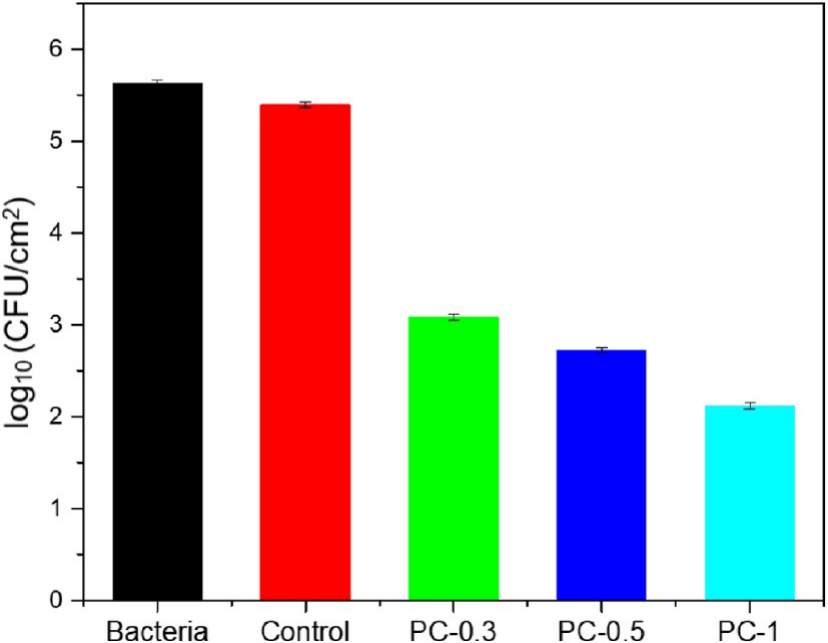
Antibacterial activity of composite specimens against S. aureus.

### Prototype Production

Prototyping was performed in order to confirm the ability to produce parts that can be used in refrigerators in the desired shape. An egg tray was prepared from a resin mixture containing 0.5 wt % AgNO_3_. The photographs of the prototype are presented in Figure 12. It took around 1 h to manufacture the prototype, followed by 20 min of postcure. It was shown that a prototype with antibacterial properties could be prepared without any loss of tensile strength.

**Figure 12:**
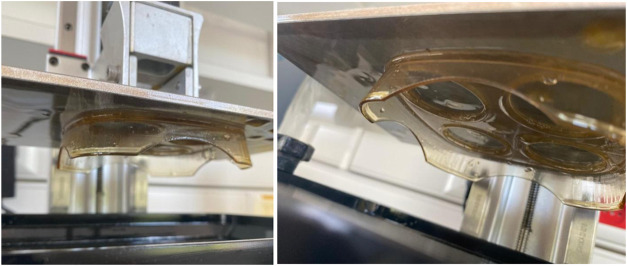
Photographs of the SLA printed nanocomposite prototype of an egg tray containing 0.5 wt % AgNO_3_.

## Conclusions

Multifunctional nanocomposites of AgNO_3_ with improved mechanical response and antibacterial activity were obtained by a lab-scale high shear mixing strategy followed by printing in a desktop-scale SLA machine. The processability, mechanical response, and antibacterial response of AgNO_3_/UV-cured resin samples having 0.1, 0.3, 0.5, 1, 3, and 5 wt % particle loadings were investigated. The processability of the liquid resin mixtures was assessed with morphological analysis focusing on the ability of the SLA machine to achieve layer-by-layer printing. SEM analysis performed on cross-sectional analysis suggested that the level of printing detail was sustained up to 0.5 wt % particle loading. UV–visible spectroscopy confirmed the formation of AgNPs during the SLA printing process without the need for an additional processing. The combinative interpretation of these results suggested that AgNO_3_ particles effectively used the provided UV energy for in situ chemical reduction while affecting the degree of polymer conversion with increasing particle loading. Such chemical reduction resulted in even darker resin mixtures as the particle amount increased beyond 1 wt %, which reduced the achievable level of detail during 3D printing. Performed EDX analysis suggested that an average agglomerate size of 400–600 nm was an approximated agglomeration size threshold causing such advertisement effects during resin processing.

The initial function of mechanical property improvement was assessed by the tensile testing of the nanocomposites. Results suggested an increasing trend in Young’s modulus with increased particle loading up to 1 wt %. A 20% improvement in *E* values was noted for PC-0.5 and PC-1 cases. A similar improvement of 20% was also noted in yield strength values of PC-0.5, whereas no improvements were noted above this particle loading. This suggested that 0.5 wt % particle loading was advantageous in terms of both stiffness and strength. Fractographic analysis performed on fractured samples suggested that the stiffening effect was achieved by effective integration of AgNPs in inherent building blocks, causing a more collective building block failure. For samples with high particle loading, such an effect was disturbed due to agglomerations.

The second function of antibacterial activity was measured according to ISO 22196. Pathogenic bacterial strains of Gram-positive S. aureus were used to evaluate the antibacterial activity by calculating the colony-forming units. Quantitative analysis based on the colony-forming units indicated that significant bacterial activity was obtained with the introduction of silver nitrate into the UV-curable resins. The demand for antibacterial activity and superior mechanical response was then met with PC-0.5.

The last effort was given to manufacturing an example egg tray with PC-0.5 resin mixture, which required 1 h of continuous printing during which no problematics in printing were noted.

The obtained results suggest that silver nitrate nanocomposites prepared via stereolithography display superior mechanical and antibacterial properties and have immense potential for use in several applications that require antibacterial activity.
